# Supplementary material for: Phylogenetic analyses of Norwegian Tenacibaculum strains confirm high bacterial diversity and suggest circulation of ubiquitous virulent strains
Source: PLoS One. 2021 Oct 28;16(10):e0259215. doi: 10.1371/journal.pone.0259215 (PMC8553039; doi:10.1371/journal.pone.0259215)
Supplement: S1 Table — Sequence type (ST) for 67 Tenacibaculum strains and unique allelic identifiers for the seven loci considered in the MLST scheme assigned by the established Tenacibaculum MLST website (https://pubmlst.org/tenacibaculum/). (DOCX) [file pone.0259215.s001.docx]

| aliases | *atpA* | *dnaK* | *glyA* | *gyrB* | *infB* | *rlmN* | *tgt* | ST |
| --- | --- | --- | --- | --- | --- | --- | --- | --- |
| LIM001 | 29 | 33 | 41 | 18 | 21 | 1005 | 1004 | ST-172 |
| LIM002 | 29 | 33 | 41 | 18 | 21 | 1005 | 1004 | ST-172 |
| LIM003 | 1007 | 1007 | 1007 | 28 | 1006 | 8 | 1005 | ST-165 |
| LIM004 | 1007 | 10 | 15 | 27 | 1007 | 1009 | 1005 | ST-96 |
| LIM005 | 29 | 1006 | 30 | 1005 | 34 | 1005 | 1004 | ST-152 |
| LIM006 | 1005 | 22 | 1005 | 25 | 21 | 1005 | 23 | ST-160 |
| LIM007 | 29 | 33 | 41 | 18 | 21 | 1005 | 1004 | ST-172 |
| LIM008 | 1006 | 1004 | 1006 | 1005 | 1004 | 1006 | 1006 | ST-162 |
| LIM009 | 29 | 33 | 41 | 18 | 21 | 1005 | 1004 | ST-172 |
| LIM010 | 1005 | 1005 | 1005 | 1004 | 1005 | 1005 | 1004 | ST-53 |
| LIM011 | 29 | 1006 | 30 | 1005 | 34 | 1005 | 1004 | ST-152 |
| LIM012 | 1007 | 11 | 1008 | 45 | 18 | 28 | 1005 | ST-118 |
| LIM013 | 29 | 1006 | 30 | 1005 | 34 | 1005 | 1004 | ST-152 |
| LIM014 | 29 | 1006 | 30 | 1005 | 34 | 1005 | 1004 | ST-152 |
| LIM016 | 1007 | 1007 | 29 | 35 | 31 | 24 | 1005 | ST-164 |
| LIM017 | 22 | 32 | 32 | 24 | 23 | 19 | 1004 | ST-143 |
| LIM018 | 1006 | 1004 | 1006 | 1005 | 1004 | 1006 | 1006 | ST-162 |
| LIM020 | 1004 | 1004 | 1004 | 1003 | 1004 | 31 | 1006 | ST-157 |
| LIM023 | 29 | 1006 | 30 | 1005 | 34 | 1005 | 1004 | ST-152 |
| LIM024 | 21 | 1010 | 1010 | 30 | 1009 | 1010 | 20 | ST-107 |
| LIM025 | 21 | 1010 | 1010 | 30 | 1009 | 1010 | 20 | ST-107 |
| LIM026 | 29 | 33 | 41 | 18 | 21 | 1005 | 1004 | ST-172 |
| LIM027 | 29 | 33 | 41 | 18 | 21 | 1005 | 1004 | ST-172 |
| LIM032 | 1004 | 1004 | 1004 | 1003 | 1004 | 1004 | 1004 | ST-52 |
| LIM033 | 29 | 1006 | 30 | 1005 | 34 | 1005 | 1004 | ST-152 |
| LIM036 | 21 | 1010 | 1010 | 30 | 1009 | 1010 | 20 | ST-107 |
| LIM040 | 29 | 33 | 41 | 18 | 21 | 1005 | 1004 | ST-172 |
| LIM042 | 29 | 1006 | 30 | 1005 | 34 | 1005 | 1004 | ST-152 |
| LIM043 | 29 | 1006 | 30 | 1005 | 34 | 1005 | 1004 | ST-152 |
| LIM044 | 29 | 33 | 41 | 18 | 21 | 1005 | 1004 | ST-172 |
| LIM046 | 29 | 33 | 41 | 18 | 21 | 1005 | 1004 | ST-172 |
| LIM047 | 1005 | 22 | 1005 | 25 | 21 | 1005 | 23 | ST-160 |
| LIM048 | 29 | 33 | 30 | 18 | 21 | 1005 | 1004 | ST-173 |
| LIM049 | 1005 | 22 | 1005 | 25 | 21 | 1005 | 23 | ST-160 |
| LIM050 | 29 | 33 | 41 | 18 | 21 | 1005 | 1004 | ST-172 |
| LIM051 | 29 | 33 | 41 | 18 | 21 | 1005 | 1004 | ST-172 |
| LIM052 | 29 | 33 | 41 | 18 | 21 | 1005 | 1004 | ST-172 |
| LIM053 | 29 | 33 | 41 | 18 | 21 | 1005 | 1004 | ST-172 |
| LIM054 | 1005 | 22 | 1005 | 23 | 21 | 1005 | 23 | ST-159 |
| LIM055 | 1005 | 22 | 1005 | 25 | 21 | 1005 | 23 | ST-160 |
| LIM056 | 1007 | 1007 | 1012 | 22 | 18 | 13 | 1005 | ST-166 |
| LIM057 | 25 | 18 | 34 | 37 | 35 | 22 | 29 | ST-144 |
| LIM058 | 26 | 19 | 26 | 46 | 37 | 17 | 30 | ST-147 |
| LIM059 | 1005 | 22 | 1005 | 25 | 21 | 1005 | 23 | ST-160 |
| LIM060 | 1005 | 22 | 1005 | 25 | 21 | 1005 | 23 | ST-160 |
| LIM061 | 29 | 1006 | 30 | 1005 | 34 | 1005 | 1004 | ST-152 |
| LIM062 | 29 | 1006 | 30 | 1005 | 34 | 1005 | 1004 | ST-152 |
| LIM063 | 21 | 1010 | 1010 | 30 | 1009 | 1010 | 20 | ST-107 |
| LIM064 | 1004 | 1007 | 1004 | 1003 | 1004 | 1004 | 1004 | ST-158 |
| LIM065 | 29 | 33 | 41 | 18 | 21 | 1005 | 1004 | ST-172 |
| LIM066 | 1007 | 1007 | 15 | 52 | 1007 | 1008 | 1005 | ST-163 |
| LIM067 | 25 | 18 | 34 | 41 | 28 | 18 | 32 | ST-145 |
| LIM068 | 1005 | 22 | 1005 | 1004 | 21 | 1005 | 3 | ST-161 |
| LIM069 | 27 | 29 | 42 | 43 | 42 | 32 | 36 | ST-148 |
| LIM070 | 28 | 26 | 25 | 50 | 22 | 16 | 28 | ST-149 |
| LIM071 | 29 | 1006 | 30 | 1005 | 34 | 1005 | 1004 | ST-152 |
| LIM072 | 25 | 18 | 36 | 40 | 25 | 23 | 33 | ST-146 |
| LIM073 | 30 | 20 | 27 | 26 | 22 | 14 | 31 | ST-153 |
| LIM074 | 29 | 33 | 41 | 18 | 21 | 1005 | 1004 | ST-172 |
| LIM075 | 31 | 21 | 33 | 31 | 43 | 27 | 34 | ST-154 |
| LIM076 | 1004 | 1004 | 1004 | 1003 | 1004 | 1004 | 1004 | ST-52 |
| LIM077 | 29 | 33 | 41 | 18 | 21 | 1005 | 1004 | ST-172 |
| LIM078 | 32 | 24 | 44 | 49 | 30 | 34 | 37 | ST-155 |
| LIM079 | 33 | 28 | 45 | 44 | 29 | 33 | 35 | ST-156 |
| LIM080 | 21 | 1010 | 1010 | 30 | 1009 | 1010 | 20 | ST-107 |
| LIM081 | 21 | 1010 | 1010 | 30 | 1009 | 1010 | 20 | ST-107 |
| strain HFJ | 39 | 1005 | 50 | 51 | 50 | 36 | 1006 | ST-174 |
