# Supplementary material for: Phylogenetic analyses of Norwegian Tenacibaculum strains confirm high bacterial diversity and suggest circulation of ubiquitous virulent strains
Source: PLoS One. 2021 Oct 28;16(10):e0259215. doi: 10.1371/journal.pone.0259215 (PMC8553039; doi:10.1371/journal.pone.0259215)
Supplement: S2 Table — (DOCX) [file pone.0259215.s002.docx]

| ID | *atpA* | *dnaK* | *glyA* | *gyrB* | *infB* | *rlmN* | *tgt* |
| --- | --- | --- | --- | --- | --- | --- | --- |
| LIM001 | MK414043 | MK414055 | MK414067 | MK414079 | MK414103 | MK414115 | MK414127 |
| LIM002 | MK414044 | MK414056 | MK414068 | MK414080 | MK414104 | MK414116 | MK414128 |
| LIM003 | MK414045 | MK414057 | MK414069 | MK414081 | MK414105 | MK414117 | MK414129 |
| LIM004 | MK414046 | MK414058 | MK414070 | MK414082 | MK414106 | MK414118 | MK414130 |
| LIM005 | MK414047 | MK414059 | MK414071 | MK414083 | MK414107 | MK414119 | MK414131 |
| LIM006 | MK414048 | MK414060 | MK414072 | MK414084 | MK414108 | MK414120 | MK414132 |
| LIM007 | MK414049 | MK414061 | MK414073 | MK414085 | MK414109 | MK414121 | MK414133 |
| LIM008 | MK414050 | MK414062 | MK414074 | MK414086 | MK414110 | MK414122 | MK414134 |
| LIM009 | MK414051 | MK414063 | MK414075 | MK414087 | MK414111 | MK414123 | MK414135 |
| LIM010 | MK414052 | MK414064 | MK414076 | MK414088 | MK414112 | MK414124 | MK414136 |
| LIM011 | MK414053 | MK414065 | MK414077 | MK414089 | MK414113 | MK414125 | MK414137 |
| LIM012 | MK414054 | MK414066 | MK414078 | MK414090 | MK414114 | MK414126 | MK414138 |
| LIM013 | MT536357 | MT536411 | MT536465 | MT536519 | MT536573 | MN688259 | MT536663 |
| LIM014 | MT536358 | MT536412 | MT536466 | MT536520 | MT536574 | MN688260 | MT536664 |
| LIM016 | MT536359 | MT536413 | MT536467 | MT536521 | MT536575 | MN688262 | MT536665 |
| LIM017 | MT536360 | MT536414 | MT536468 | MT536522 | MT536576 | MN688263 | MT536666 |
| LIM018 | MT536361 | MT536415 | MT536469 | MT536523 | MT536577 | MN688264 | MT536667 |
| LIM020 | MT536362 | MT536416 | MT536470 | MT536524 | MT536578 | MN688266 | MT536668 |
| LIM023 | MT536363 | MT536417 | MT536471 | MT536525 | MT536579 | MN688269 | MT536669 |
| LIM024 | MT536364 | MT536418 | MT536472 | MT536526 | MT536580 | MN688270 | MT536670 |
| LIM025 | MT536365 | MT536419 | MT536473 | MT536527 | MT536581 | MN688271 | MT536671 |
| LIM026 | MT536366 | MT536420 | MT536474 | MT536528 | MT536582 | MN688272 | MT536672 |
| LIM027 | MT536367 | MT536421 | MT536475 | MT536529 | MT536583 | MN688273 | MT536673 |
| LIM032 | MT536368 | MT536422 | MT536476 | MT536530 | MT536584 | MN688278 | MT536674 |
| LIM033 | MT536369 | MT536423 | MT536477 | MT536531 | MT536585 | MN688279 | MT536675 |
| LIM036 | MT536370 | MT536424 | MT536478 | MT536532 | MT536586 | MN688282 | MT536676 |
| LIM040 | MT536371 | MT536425 | MT536479 | MT536533 | MT536587 | MN688286 | MT536677 |
| LIM042 | MT536372 | MT536426 | MT536480 | MT536534 | MT536588 | MN688288 | MT536678 |
| LIM043 | MT536373 | MT536427 | MT536481 | MT536535 | MT536589 | MN688289 | MT536679 |
| LIM044 | MT536374 | MT536428 | MT536482 | MT536536 | MT536590 | MN688290 | MT536680 |
| LIM046 | MT536375 | MT536429 | MT536483 | MT536537 | MT536591 | MT536627 | MT536681 |
| LIM047 | MT536376 | MT536430 | MT536484 | MT536538 | MT536592 | MT536628 | MT536682 |
| LIM048 | MT536377 | MT536431 | MT536485 | MT536539 | MT536593 | MT536629 | MT536683 |
| LIM049 | MT536378 | MT536432 | MT536486 | MT536540 | MT536594 | MT536630 | MT536684 |
| LIM050 | MT536379 | MT536433 | MT536487 | MT536541 | MT536595 | MT536631 | MT536685 |
| LIM051 | MT536380 | MT536434 | MT536488 | MT536542 | MT536596 | MT536632 | MT536686 |
| LIM052 | MT536381 | MT536435 | MT536489 | MT536543 | MT536597 | MT536633 | MT536687 |
| LIM053 | MT536382 | MT536436 | MT536490 | MT536544 | MT536598 | MT536634 | MT536688 |
| LIM054 | MT536383 | MT536437 | MT536491 | MT536545 | MT536599 | MT536635 | MT536689 |
| LIM055 | MT536384 | MT536438 | MT536492 | MT536546 | MT536600 | MT536636 | MT536690 |
| LIM056 | MT536385 | MT536439 | MT536493 | MT536547 | MT536601 | MT536637 | MT536691 |
| LIM057 | MT536386 | MT536440 | MT536494 | MT536548 | MT536602 | MT536638 | MT536692 |
| LIM058 | MT536387 | MT536441 | MT536495 | MT536549 | MT536603 | MT536639 | MT536693 |
| LIM059 | MT536388 | MT536442 | MT536496 | MT536550 | MT536604 | MT536640 | MT536694 |
| LIM060 | MT536389 | MT536443 | MT536497 | MT536551 | MT536605 | MT536641 | MT536695 |
| LIM061 | MT536390 | MT536444 | MT536498 | MT536552 | MT536606 | MT536642 | MT536696 |
| LIM062 | MT536391 | MT536445 | MT536499 | MT536553 | MT536607 | MT536643 | MT536697 |
| LIM063 | MT536392 | MT536446 | MT536500 | MT536554 | MT536608 | MT536644 | MT536698 |
| LIM064 | MT536393 | MT536447 | MT536501 | MT536555 | MT536609 | MT536645 | MT536699 |
| LIM065 | MT536394 | MT536448 | MT536502 | MT536556 | MT536610 | MT536646 | MT536700 |
| LIM066 | MT536395 | MT536449 | MT536503 | MT536557 | MT536611 | MT536647 | MT536701 |
| LIM067 | MT536396 | MT536450 | MT536504 | MT536558 | MT536612 | MT536648 | MT536702 |
| LIM068 | MT536397 | MT536451 | MT536505 | MT536559 | MT536613 | MT536649 | MT536703 |
| LIM069 | MT536398 | MT536452 | MT536506 | MT536560 | MT536614 | MT536650 | MT536704 |
| LIM070 | MT536399 | MT536453 | MT536507 | MT536561 | MT536615 | MT536651 | MT536705 |
| LIM071 | MT536400 | MT536454 | MT536508 | MT536562 | MT536616 | MT536652 | MT536706 |
| LIM072 | MT536401 | MT536455 | MT536509 | MT536563 | MT536617 | MT536653 | MT536707 |
| LIM073 | MT536402 | MT536456 | MT536510 | MT536564 | MT536618 | MT536654 | MT536708 |
| LIM074 | MT536403 | MT536457 | MT536511 | MT536565 | MT536619 | MT536655 | MT536709 |
| LIM075 | MT536404 | MT536458 | MT536512 | MT536566 | MT536620 | MT536656 | MT536710 |
| LIM076 | MT536405 | MT536459 | MT536513 | MT536567 | MT536621 | MT536657 | MT536711 |
| LIM077 | MT536406 | MT536460 | MT536514 | MT536568 | MT536622 | MT536658 | MT536712 |
| LIM078 | MT536407 | MT536461 | MT536515 | MT536569 | MT536623 | MT536659 | MT536713 |
| LIM079 | MT536408 | MT536462 | MT536516 | MT536570 | MT536624 | MT536660 | MT536714 |
| LIM080 | MT536409 | MT536463 | MT536517 | MT536571 | MT536625 | MT536661 | MT536715 |
| LIM081 | MT536410 | MT536464 | MT536518 | MT536572 | MT536626 | MT536662 | MT536716 |
