# Supplementary material for: Phylogenetic analyses of Norwegian Tenacibaculum strains confirm high bacterial diversity and suggest circulation of ubiquitous virulent strains
Source: PLoS One. 2021 Oct 28;16(10):e0259215. doi: 10.1371/journal.pone.0259215 (PMC8553039; doi:10.1371/journal.pone.0259215)
Supplement: S3 Table — Growth characteristics on blood Marine Agar of the 29 sequence types isolated in the study. (DOCX) [file pone.0259215.s003.docx]

| ST | Colony caracteristics (on BAMA) |
| --- | --- |
| ST-52 | Haemolytic, yellow, slightly iridescent, sticky. Long and thin bacteria |
| ST-53 | Non-haemolytic, yellow |
| ST-96 | Non-haemolytic, yellow pale |
| ST-107 | Very haemolytic, yellow, greenish iridescent after 2 days. Very long bacteria |
| ST-118 | Non-haemolytic, yellow, medium sticky. Long bacteria |
| ST-143 | Non-haemolytic, yellow |
| ST-144 | Non-haemolytic, yellow |
| ST-145 | Non-haemolytic, yellow, slightly sticky |
| ST-146 | Non-haemolytic, yellow-white, slightly rugous |
| ST-147 | Haemolytic, yellow |
| ST-148 | Non-haemolytic, bright yellow |
| ST-149 | Haemolytic, pinkish, highly iridescent |
| ST-152 | Haemolytic, yellow, turning yellow-pink with iridescence after 2 days of growth. Medium-sized bacteria |
| ST-153 | Haemolytic, white |
| ST-154 | Non-haemolytic, bright yellow. Very long bacteria |
| ST-155 | Non-haemolytic, purple with iridescence |
| ST-156 | Non-haemolytic, yellow-green with iridescence |
| ST-157 | Haemolytic, yellow |
| ST-158 | Haemolytic, pale yellow, very sticky |
| ST-159 | Non-haemolytic, yellow |
| ST-160 | Non-haemolytic, yellow, with slight purple iridescence |
| ST-161 | Non-haemolytic, yellow-white |
| ST-162 | Haemolytic, yellow-white, turning more pink after several days of growth |
| ST-162 | Haemolytic, yellow-white |
| ST-163 | Non-haemolytic, yellow-pink with iridescence |
| ST-164 | Non-haemolytic, white |
| ST-165 | Non-haemolytic, yellow pale |
| ST-166 | Non-haemolytic, yellow pale |
| ST-172 | Haemolytic, yellow pale colony, slightly sticky. Long filamentous bacteria |
