# Supplementary figures and images for: Phylogenetic analyses of Norwegian Tenacibaculum strains confirm high bacterial diversity and suggest circulation of ubiquitous virulent strains
Source: PLoS One. 2021 Oct 28;16(10):e0259215. doi: 10.1371/journal.pone.0259215 (PMC8553039; doi:10.1371/journal.pone.0259215)

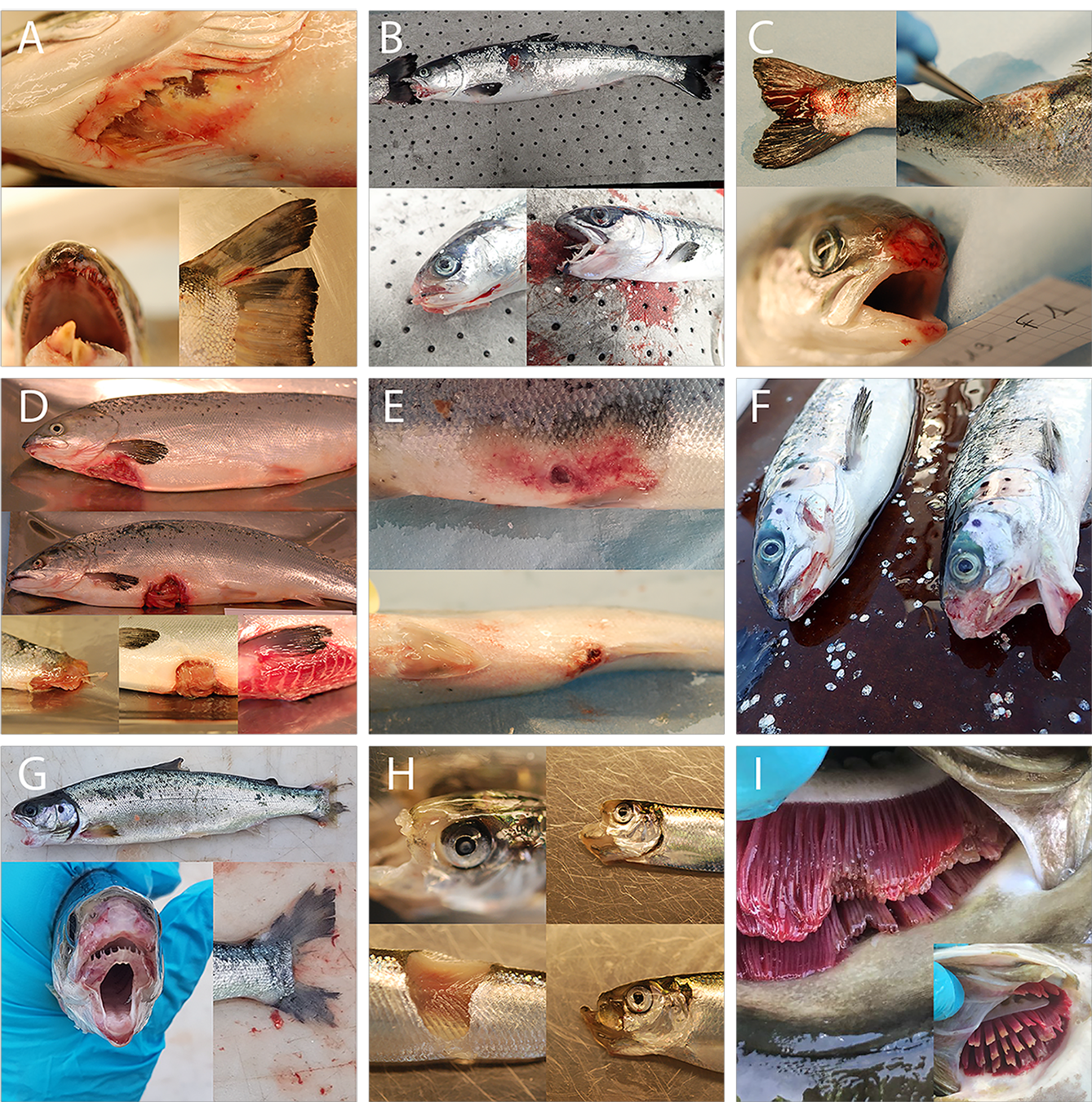

Supplement: S1 Fig — A: sampling 1. B: sampling 9. C: sampling 11. D: sampling 12. E: sampling 14. F: sampling 15. G: sampling 21. H: sampling 27. I: sampling 28. Image credits: Photo by Erwan Lagadec (C, I), MarinHelse AS (B, F, G), Heidrun Nylund (A, D, E), Kjetil Solheim (H). (TIF) [file pone.0259215.s005.tif]
