# Supplementary material for: Phylogenetic analyses of Norwegian Tenacibaculum strains confirm high bacterial diversity and suggest circulation of ubiquitous virulent strains
Source: PLoS One. 2021 Oct 28;16(10):e0259215. doi: 10.1371/journal.pone.0259215 (PMC8553039; doi:10.1371/journal.pone.0259215)
Supplement: Attachment — Submitted filename: PONE-D-21-21158.docx [file pone.0259215.s006.docx]

#### PLOS ONE

**Phylogenetic analyses of Norwegian Tenacibaculum strains confirm high bacterial diversity and suggest circulation of ubiquitous virulent strains**

--Manuscript Draft--

| **Manuscript Number:** | PONE-D-21-21158 |
| --- | --- |
| **Article Type:** | Research Article |
| **Full Title:** | Phylogenetic analyses of Norwegian Tenacibaculum strains confirm high bacterial diversity and suggest circulation of ubiquitous virulent strains |
| **Short Title:** | Phylogenetic analyses of Norwegian Tenacibaculum strains |
| **Corresponding Author:** | Erwan Lagadec  University of Bergen: Universitetet i Bergen Bergen, NORWAY |
| **Keywords:** | Tenacibaculosis; Tenacibaculum; Salmo salar, ulcerative disease; Fish disease; MLSA; MLST |
| **Abstract:** | Tenacibaculosis is a bacterial ulcerative disease affecting marine fish and represents a major threat to aquaculture worldwide. Its aetiological agents, bacteria belonging to the genus Tenacibaculum, have been present in Norway since the late 1980’s and lead to regular ulcerative outbreaks and high mortalities in production of farmed salmonids.  Studies have shown the presence of several Tenacibaculum species in Norway and a lack of clonality in outbreak-related strains, thus preventing the development of an effective vaccine. Hence, a thorough examination of the bacterial diversity in farmed fish presenting ulcers and the geographical distribution of the pathogens should provide important insights needed to strengthen preventive actions. In this study, we investigated the diversity of Tenacibaculum strains isolated in 28 outbreaks that occurred in Norwegian fish farms in the period 2017-2020. We found that 95% of the 66 strains isolated and characterized, using an existing MultiLocus Sequence Typing system, have not previously been identified, confirming the high diversity of this genus of bacteria in Norway. Several of these Tenacibaculum species seem to be present within restricted areas (e.g. Tenacibaculum dicentrarchi in western Norway), but phylogenetic analysis reveals that several of the strains responsible of ulcerative outbreaks were isolated from different localities (e.g. ST- 172 isolated from northern to southern parts of Norway) and/or from different hosts. Understanding their reservoirs and transmission pathways could help to address major challenges in connection with prophylactic measures and development of vaccines. |
| **Order of Authors:** | Erwan Lagadec |
|  | Sverre Bang Småge |
|  | Christiane Trösse |
|  | Are Nylund |
| **Additional Information:** |  |
| **Question** | **Response** |
| **Financial Disclosure**  Enter a financial disclosure statement that describes the sources of funding for the work included in this submission. Review the [submission guidelines](http://journals.plos.org/plosone/s/submission-guidelines#loc-financial-disclosure-statement) for detailed requirements. View published research articles from [*PLOS ONE*](http://journals.plos.org/plosone/) for specific examples.  This statement is required for submission | This study was supported by a FHF grant (Fiskeri og Havbruksnæringens Forskningsfond, LimiT project #901433). The grant was received by AN. The funders had no role in study design, data collection and analysis, decision to publish or preparation of the manuscript. |

| and **will appear in the published article** if the submission is accepted. Please make sure it is accurate. | |  |
| --- | --- | --- |
| **Unfunded studies**  Enter: *The author(s) received no specific funding for this work.*  **Funded studies**  Enter a statement with the following details:   - Initials of the authors who received each award - Grant numbers awarded to each author - The full name of each funder - URL of each funder website - Did the sponsors or funders play any role in the study design, data collection and analysis, decision to publish, or preparation of the manuscript? - **NO** - Include this sentence at the end of your statement: *The funders had no role in study design, data collection and analysis, decision to publish, or preparation of the manuscript.* - **YES** - Specify the role(s) played. | |  |
| * typeset | |  |
| **Competing Interests**  Use the instructions below to enter a competing interest statement for this submission. On behalf of all authors, disclose any [competing interests](http://journals.plos.org/plosone/s/competing-interests) that could be perceived to bias this work—acknowledging all financial support and any other relevant financial or non- financial competing interests.  This statement is required for submission and **will appear in the published article** if the submission is accepted. Please make sure it is accurate and that any funding sources listed in your Funding Information later in the submission form are also declared in your Financial Disclosure statement.  View published research articles from  [*PLOS ONE*](http://journals.plos.org/plosone/) for specific examples. | | The authors have declared that no competing interests exist. |
|  |  |  |

| **NO authors have competing interests**  Enter: *The authors have declared that no competing interests exist.*  **Authors with competing interests**  Enter competing interest details beginning with this statement:  *I have read the journal's policy and the authors of this manuscript have the following competing interests: [insert competing interests here]* |  |
| --- | --- |
| * typeset |  |
| **Ethics Statement**  Enter an ethics statement for this submission. This statement is required if the study involved:   - Human participants - Human specimens or tissue - Vertebrate animals or cephalopods - Vertebrate embryos or tissues - Field research   Write "N/A" if the submission does not require an ethics statement.  General guidance is provided below. Consult the [submission guidelines](http://journals.plos.org/plosone/s/submission-guidelines#loc-guidelines-for-specific-study-types) for detailed instructions. **Make sure that all information entered here is included in the Methods section of the manuscript.** | Fish samples were collected from licensed Norwegian fish farms (Norwegian Directorate of Fisheries). The fish were treated by veterinarians and aqua-medicine biologists according to the Norwegian Animal Welfare Act (01.01.2010) and the study strictly followed the regulations set by the Norwegian Food Safety Authority. |

| **Format for specific study types**  **Human Subject Research (involving human participants and/or tissue)**   - Give the name of the institutional review board or ethics committee that approved the study - Include the approval number and/or a statement indicating approval of this research - Indicate the form of consent obtained (written/oral) or the reason that consent was not obtained (e.g. the data were analyzed anonymously)   **Animal Research (involving vertebrate animals, embryos or tissues)**   - Provide the name of the Institutional Animal   Care and Use Committee (IACUC) or other relevant ethics board that reviewed the study protocol, and indicate whether they approved this research or granted a formal waiver of ethical approval   - Include an approval number if one was obtained - If the study involved *non-human primates*, add *additional details* about animal welfare and steps taken to ameliorate suffering - If anesthesia, euthanasia, or any kind of animal sacrifice is part of the study, include briefly which substances and/or methods were applied   **Field Research**  Include the following details if this study involves the collection of plant, animal, or other materials from a natural setting:   - Field permit number - Name of the institution or relevant body that granted permission | |  |
| --- | --- | --- |
|  | |  |
| **Data Availability**  Authors are required to make all data underlying the findings described fully available, without restriction, and from the time of publication. PLOS allows rare exceptions to address legal and ethical concerns. See the [PLOS Data Policy](http://journals.plos.org/plosone/s/data-availability) and [FAQ](http://journals.plos.org/plosone/s/data-availability#loc-faqs-for-data-policy) for detailed information. | | Yes - all data are fully available without restriction |
|  |  |  |

| A Data Availability Statement describing where the data can be found is required at submission. Your answers to this question constitute the Data Availability Statement and **will be published in the article**, if accepted. |  |
| --- | --- |
| **Important:** Stating ‘data available on request from the author’ is not sufficient. If your data are only available upon request, select ‘No’ for the first question and explain your exceptional situation in the text box. |  |
| Do the authors confirm that all data underlying the findings described in their manuscript are fully available without restriction? |  |
| **Describe where the data may be found in full sentences. If you are copying our sample text, replace any instances of XXX with the appropriate details.**   - If the data are **held or will be held in a public repository**, include URLs, accession numbers or DOIs. If this information will only be available after acceptance, indicate this by ticking the box below. For example: *All XXX files are available from the XXX database (accession number(s) XXX, XXX.*). - If the data are all contained **within the manuscript and/or Supporting Information files**, enter the following: *All relevant data are within the manuscript and its Supporting Information files.* - If neither of these applies but you are able to provide **details of access elsewhere**, with or without limitations, please do so. For example:   *Data cannot be shared publicly because of [XXX]. Data are available from the XXX Institutional Data Access / Ethics Committee (contact via XXX) for researchers who meet the criteria for access to confidential data.*  *The data underlying the results presented in the study are available from (include the name of the third party* | All relevant data are within the manuscript and its Supporting Information files. All nucleotide sequences are available from the GenBank database [(https://www.ncbi.nlm.nih.gov/genbank/).](http://www.ncbi.nlm.nih.gov/genbank/)) All accession numbers are listed in Supporting Information S2. |

| *and contact information or URL).*   - This text is appropriate if the data are owned by a third party and authors do not have permission to share the data.   * typeset |  |
| --- | --- |
| Additional data availability information: | Tick here if the URLs/accession numbers/DOIs will be available only after acceptance of the manuscript for publication so that we can ensure their inclusion before publication. |

Manuscript	[Click here to access/download;Manuscript;Manuscript.docx](https://www.editorialmanager.com/pone/download.aspx?id=29338879&amp;guid=f9ae2379-2800-4c0b-a3c0-fb2e2798a94b&amp;scheme=1)

1 Phylogenetic analyses of Norwegian *Tenacibaculum* strains confirm high bacterial
2 **diversity and suggest circulation of ubiquitous virulent strains.**

3

4	Erwan Lagadec^1*^, Sverre Bang Småge^2^, Christiane Trösse^1^, Are Nylund^1^

5

6 1 Fish Disease Research Group, Department of Biological Sciences, University of Bergen,
7 Bergen, Norway
8 2 Cermaq Group AS, Oslo, Norway

9

10 * Corresponding author
11 Erwan Lagadec
12 E-[mail : erwan.lagadec@uib.no](mailto:erwan.lagadec@uib.no) (EL)

### 13 Abstract

14 Tenacibaculosis is a bacterial ulcerative disease affecting marine fish and represents a major
15 threat to aquaculture worldwide. Its aetiological agents, bacteria belonging to the genus
16 *Tenacibaculum*, have been present in Norway since the late 1980’s and lead to regular
17 ulcerative outbreaks and high mortalities in production of farmed salmonids. Studies have
18 shown the presence of several *Tenacibaculum* species in Norway and a lack of clonality in
19 outbreak-related strains, thus preventing the development of an effective vaccine. Hence, a
20 thorough examination of the bacterial diversity in farmed fish presenting ulcers and the
21 geographical distribution of the pathogens should provide important insights needed to
22 strengthen preventive actions. In this study, we investigated the diversity of *Tenacibaculum*
23 strains isolated in 28 outbreaks that occurred in Norwegian fish farms in the period 2017-2020.
24 We found that 95% of the 66 strains isolated and characterized, using an existing MultiLocus
25 Sequence Typing system, have not previously been identified, confirming the high diversity of
26 this genus of bacteria in Norway. Several of these *Tenacibaculum* species seem to be present
27 within restricted areas (e.g. *Tenacibaculum dicentrarchi* in western Norway), but phylogenetic
28 analysis reveals that several of the strains responsible of ulcerative outbreaks were isolated from
29 different localities (e.g. ST- 172 isolated from northern to southern parts of Norway) and/or
30 from different hosts. Understanding their reservoirs and transmission pathways could help to
31 address major challenges in connection with prophylactic measures and development of
32 vaccines.

33

34

### 35 Introduction

36 Tenacibaculosis, caused by members of the genus *Tenacibaculum,* is an ulcerative skin disease
37 of many economically important marine farmed fish species worldwide. This disease is of major
38 concern for global fish production and is currently considered one of the most threatening
39 bacterial disease impacting mariculture [1]. Tenacibaculosis is associated with characteristic
40 clinical signs such as ulcerative skin lesions, mouth erosion, frayed fins, and tail. The best-
41 known pathogen in the genus is the type species *Tenacibaculum maritimum* which has been
42 reported from disease outbreaks in farmed fish since the 1970s [2-7]. However, in recent years
43 an increasing number of novel *Tenacibaculum* species have been isolated and associated with
44 ulcerative skin disease in farmed marine fishes [8-14]]. Tenacibaculosis is reported from marine
45 wild and farmed fish in Europe, Asia, North and South America and Australia [2, 4, 5, 9, 15-

46	18].

47 The first time a *Tenacibaculum* species was associated with fish disease in Norway was in 1989
48 with the novel bacterium *Tenacibaculum ovolyticum.* This bacterium was isolated from the
49 adherent epiflora of halibut eggs and was shown to be an opportunistic pathogen for halibut egg
50 and larvae [9, 19]. Since then, an increasing number of novel *Tenacibaculum* spp. have been
51 associated with ulcerative skin disease in, or shown to be pathogenic to, farmed marine fish in
52 Norway[11, 14, 20-24]. Due to the increased use of Marine Agar (MA), for bacterial isolation in the
53 recent years *Tenacibaculum* spp. have also been associated with the winter ulcer disease
54 previously attributed to the bacterium *Moritella viscosa* [25, 26]. From phylogenetic analyses
55 it has been shown that there is a large *Tenacibaculum* spp. diversity in Norway [11, 14, 21, 23,
56 27, 28]. However, most *Tenacibaculum* isolates associated with ulcerative disease belong to
57 *Tenacibaculum	finnmarkense*	genomovar	*finnmarkense*,	*Tenacibaculum	finnmarkense*
58 genomovar *ulcerans* and *Tenacibaculum dicentrarchi* [11, 23, 27].
59 Through a MultiLocus Sequence Analysis, the present study aimed at investigating the
60 *Tenacibaculum* spp. diversity associated with outbreaks of ulcerative disease in Norwegian fish
61 farms for the period 2017-2020 with an emphasis on the northern part, where the aquaculture
62 industry suffers the most from tenacibaculosis. By expanding the analysis to *Tenacibaculum*
63 spp. isolated all along the Norwegian coast, the presence of ubiquitous or multi-hosts
64 *Tenacibaculum* strains that could be of importance in terms of future epidemic management of
65 tenacibaculosis in salmon production was explored.

### 66 Material and Methods

#### 67 Bacteria isolation

68 Fish samples were collected from licensed Norwegian fish farms (Norwegian Directorate of
69 Fisheries). The fish were treated by veterinarians and aqua-medicine biologists according to
70 the Norwegian Animal Welfare Act (01.01.2010) and the study strictly followed the
71 regulations set by the Norwegian Food Safety Authority.
72 Farmed marine fishes (Atlantic herring (*Clupea harengus*), Atlantic halibut (*Hippoglossus*
73 *hippoglossus*), Atlantic salmon (*Salmo salar*), lumpfish (*Cyclopterus lumpus)* and rainbow
74 trout (*Oncorhynchus mykiss*)) suffering ulcerative skin disease in Norway were sampled in the
75 period from 2017 to 2019. Bacteria were isolated from affected tissues (i.e., gills, skin, mouth,
76 fins and from internal organs), on Marine Agar (DIFCO 2216), on blood marine agar (BAMA),
77 or on BAMA supplemented with kanamycin to a final concentration of 50 µg/ml.
78 Bacteria isolated from field samples were cultured for a minimum of three days at 16 °C. Plates
79 were visually checked for the presence of *Tenacibaculum* spp. by looking for characteristic
80 colonies as described for *Tenacibaculum* spp. [7, 9, 19]. The colonies were further examined
81 by using a light microscope to identify the typical *Tenacibaculum* spp. bacterial cell
82 morphology [19]. Colonies containing bacteria with *Tenacibaculum* spp. morphology were
83 further sub-cultured onto marine agar and incubated for two days at 16 °C or 20 °C. Clonal
84 *Tenacibaculum* spp. cultures were stored in 50:50 Biofreeze freezing medium (Biochrom™,
85 Germany) and Marine broth (DIFCO 2216) in liquid nitrogen.

#### 86 DNA extraction, 16S rRNA PCR and sequencing

87 Genomic DNA was extracted from the recovered *Tenacibaculum* isolates either by using a
88 DNeasy Blood & Tissue kit (Qiagen) or by heating single colonies in nuclease-free water at 96
89 °C for 5 min. The heated colonies were subsequently centrifuged at 10,000 rpm for 5 min and
90 the DNA containing supernatant transferred into new vials. All genomic DNA were stored at -

91	20 °C.

92 PCR and sequencing of the 16S rRNA gene were performed on all isolates using the primers
93 27F and 1518R [29]. Amplification details are given in [17]. PCR products were deposited on
94 a 1 % agarose electrophoresis gel stained with GelRed™ (Biotium, USA). Obtained amplicons
95 were purified using ExoCleanUp FAST (VWR) in a Veriti thermal cycler (Applied Biosystems)
96 at 37 °C for 5 min and 80 °C for 10 min before being sequenced at the Sequencing Facility at
97 the University of Bergen (<http://www.uib.no/seqlab>) using the BigDye Terminator v3.1 Cycle
98 Sequencing Kit (Applied Biosystems).
99 Consensus sequences from each sample were obtained using VectorNTI 9.0.0 software

100

(Invitrogen) and a BLAST search [30] was performed for preliminary bacterial identification.

101

#### Genetic characterization

102

Bacteria confirmed to be *Tenacibaculum* spp. from 16S rRNA gene sequence analyses were

103

further included in a MLST analysis using primers targeting seven housekeeping genes *atpA*

104

(567 bp), *dnaK* (573 bp), *glyA* (558 bp), *gyrB* (597 bp), *infB* (564 bp), *rlmN* (549 bp) and *tgt*

105

(486 bp) as previously described [28].

106

The established *Tenacibaculum* MLST website (https://pubmlst.org/tenacibaculum/) assigned

107

unique allele identifiers for the seven loci considered and defined corresponding allelic profiles

108

referred to as sequence types (STs) (S1 Table). In order to determine the DNA relatedness

109

among *Tenacibaculum* strains, a minimum-spanning tree based on the seven loci was generated

110

in	PHYLOViZ	2.0	using	the	goeBURST	Full	MST	algorithm

111

(<https://online.phyloviz.net/index>) [31, 32].

112

MultiLocus Sequence Analysis (MLSA) was conducted to compare the *Tenacibaculum* spp*.*

113

strains included in this study with strains previously described in other studies. The analysis

114

included	sequences	of	the	following	additional	*Tenacibaculum*	spp.: *Tenacibaculum*

115

*finnmarkense* genomovar *finnmarkense* strain HFJ [14], and 36 sequences obtained from

116

GenBank: 15 *Tenacibaculum* spp. strains isolated in Norway (TNO) and 21 type strains in the

117

genus *Tenacibaculum* (*T. adriaticum*T, *T. aestuarii*T, *T. aiptasiae*T, *T. amylolyticum*T, *T.*

118

*crassostreae*T, *T. discolor*T, *T. dicentrarchi*T, *T. finnmarkense* genomovar *finnmarkense*T, *T.*

119

*finnmarkense* genomovar *ulcerans*T, *T. gallaicum*T, *T. geojense*T, *T. jejuense*T, *T. litopenaei*T, *T.*

120

*litoreum*T, *T. lutimaris*T, *T. maritimum*T, *T. mesophilum*T, *T. ovolyticum*T, *T. piscium*T, *T.*

121

*skagerrakense*T, *T. soleae*T). In accordance with [28], *Kordia algicida* (Accession number

122

NZ_DS544873.1) was used as an outgroup.

123

Sequence alignments were constructed for all seven loci separately using AlignX in the

124

VectorNTI 9.0.0 software package (Invitrogen). The sequences were trimmed and adjusted to

125

correct reading frames in GeneDoc [33]. Concatenation of the seven housekeeping genes was

126

performed using Kakusan4 [34]. The substitution rate, codon position and best fit model for the

127

individual loci were calculated with Kakusan4. The Bayesian phylogenetic analysis was

128

performed in MrBayes 3.2 [35] with a proportional codonproportional data bloc and a Markov

129

Chain Monte Carlo (MCMC) analysis. The run included 20,000,000 generations and trees were

130

sampled every 1000 generations. The initial 10,000 trees were discarded as a conservative

131

“burn-in”	in	TreeAnnotator	and	the	final	tree	was	visualized	in	FigTree	v1.4.3

132

(<http://tree.bio.ed.ac.uk/>). GenBank accession numbers of the sequences from this study are

133

provided in S2 Table.

134

### Results

135

#### Isolation of bacteria from diseased fish

136

In the present study, more than 300 *Tenacibaculum* spp. isolates were recovered from five fish

137

species: Atlantic herring (*Clupea harengus*), Atlantic halibut (*Hippoglossus hippoglossus*),

138

Atlantic	salmon	(*Salmo	salar*),	lumpfish	(*Cyclopterus	lumpus)*	and	rainbow	trout

139

(*Oncorhynchus mykiss*). 28 outbreaks in fish farms were investigated in eight counties along

140

the Norwegian coast (Akershus, Finnmark, Hordaland, Møre og Romsdal, Nordland, Rogaland,

141

Troms and Sogn og Fjordane). We successfully amplified and sequenced seven MLST loci

142

from 66 strains isolated from the 28 outbreaks (Table 1).

143

Table 1. Data associated with the 66 *Tenacibaculum* strains isolated during this study.

| ID | Country | Region Sampling number | Date of Fish host β-haemolytic  collection activity Organ isolation | |
| --- | --- | --- | --- | --- |
| LIM001 LIM002 LIM003 LIM004 | Norway Norway Norway Norway | Sogn og F.^*^ 1  Sogn og F.^*^ 1  Sogn og F.^*^ 1  Sogn og F.^*^ 1 | apr-2018 *Salmo salar* yes  apr-2018 *Salmo salar* yes  apr-2018 *Salmo salar* no  apr-2018 *Salmo salar* no | skin ulcer skin ulcer skin ulcer skin ulcer |
| LIM005 | Norway | Troms 2 | apr-2018 *Salmo salar* yes | skin ulcer |
| LIM006 | Norway | Troms 2 | apr-2018 *Salmo salar* no | skin ulcer |
| LIM007 | Norway | Troms 2 | apr-2018 *Salmo salar* yes | skin ulcer |
| LIM008 | Norway | Troms 2 | apr-2018 *Salmo salar* yes | skin ulcer |
| LIM009 | Norway | Troms 2 | apr-2018 *Salmo salar* yes | skin ulcer |
| LIM010 | Norway | Troms 3 | apr-2018 *Salmo salar* no | skin ulcer |
| LIM011 | Norway | Troms 5 | sep-2019 *Salmo salar* yes | skin ulcer |
| LIM012 | Norway | Troms 5 | sep-2019 *Salmo salar* no | skin ulcer |
| LIM013 | Norway | Hordaland 6 | apr-2017 *Salmo salar* yes | skin ulcer |
| LIM014 | Norway | Hordaland 6 | apr-2017 *Salmo salar* yes | skin ulcer |

| LIM016 | Norway | Troms | 2 | apr-2018 | *Salmo salar* | no | skin ulcer |
| --- | --- | --- | --- | --- | --- | --- | --- |
| LIM017 | Norway | Troms | 2 | apr-2018 | *Salmo salar* | no | skin ulcer |
| LIM018 | Norway | Troms | 3 | apr-2018 | *Salmo salar* | yes | skin ulcer |
| LIM020 | Norway | Troms | 4 | apr-2018 | *Salmo salar* | yes | skin ulcer |
| LIM023 | Norway | Troms | 7 | apr-2018 | *Salmo salar* | yes | skin ulcer |
| LIM024 | Norway | Hordaland | 8 | mar-2019 | *Salmo salar* | yes | skin ulcer |
| LIM025 | Norway | Hordaland | 8 | mar-2019 | *Salmo salar* | yes | skin ulcer |
| LIM026 | Norway | Nordland | 9 | mar-2019 | *Salmo salar* | yes | skin ulcer |
| LIM027 | Norway | Nordland | 9 | mar-2019 | *Salmo salar* | yes | head ulcer |
| LIM032 | Norway | Akershus | 10 | mar-2019 | *Salmo salar* | yes | gill |
| LIM033 | Norway | Akershus | 10 | mar-2019 | *Salmo salar* | yes | gill |
| LIM036 | Norway | Hordaland | 11 | apr-2019 | *Salmo salar* | yes | mouth, skin ulcer |
| LIM040 | Norway | Nordland | 12 | apr-2019 | *Salmo salar* | yes | skin ulcer |
| LIM042 | Norway | Hordaland | 13 | apr-2019 | *Salmo salar* | yes | mouth, gill |
| LIM043 | Norway | Hordaland | 14 | apr-2019 | *Salmo salar* | yes | mouth, gill |
| LIM044 | Norway | Troms | 15 | apr-2019 | *Salmo salar* | yes | mouth ulcer |
| LIM046 | Norway | Troms | 2 | apr-2018 | *Salmo salar* | yes | kidney |
| LIM047 | Norway | Troms | 2 | apr-2018 | *Salmo salar* | no | kidney |
| LIM048 | Norway | Troms | 2 | apr-2018 | *Salmo salar* | yes | kidney |
| LIM049 | Norway | Troms | 2 | apr-2018 | *Salmo salar* | no | skin ulcer |
| LIM050 | Norway | Troms | 2 | apr-2018 | *Salmo salar* | yes | skin ulcer |
| LIM051 | Norway | Troms | 2 | apr-2018 | *Salmo salar* | yes | skin ulcer |
| LIM052 | Norway | Troms | 2 | apr-2018 | *Salmo salar* | yes | skin ulcer |
| LIM053 | Norway | Troms | 2 | apr-2018 | *Salmo salar* | yes | skin ulcer |
| LIM054 | Norway | Troms | 3 | apr-2018 | *Salmo salar* | no | skin ulcer |
| LIM055 | Norway | Troms | 3 | apr-2018 | *Salmo salar* | no | skin ulcer |
| LIM056 | Norway | Hordaland | 16 | oct-2017 | *Salmo salar* | no | mouth |
| LIM057 | Norway | Hordaland | 16 | oct-2017 | *Salmo salar* | no | mouth |
| LIM058 | Norway | Hordaland | 16 | oct-2017 | *Salmo salar* | yes | mouth |
| LIM059 | Norway | Rogaland | 7 | apr-2018 | *Salmo salar* | no | kidney |
| LIM060 | Norway | Rogaland | 7 | apr-2018 | *Salmo salar* | no | skin ulcer |
| LIM061 | Norway | Hordaland | 6 | apr-2017 | *Salmo salar* | yes | mouth |
| LIM062 | Norway | Troms | 11 | apr-2019 | *Salmo salar* | yes | mouth, gill |
| LIM063 | Norway | Troms | 17 | nov-2019 | *Salmo salar* | yes | skin ulcer |
| LIM064 | Norway | Hordaland | 18 | mar-2019 | *Hippoglossus*  *hippoglossus* | yes | skin ulcer |
| LIM065 | Norway | Hordaland | 19 | apr-2019 | *Hippoglossus hippoglossus* | yes | skin ulcer |
| LIM066 | Norway | Hordaland | 20 | apr-2019 | *Cyclopterus*  *lumpus* | no | skin ulcer, eye |
| LIM067 | Norway | Sogn og F.^*^ | 21 | mar-2019 | *Salmo salar* | no | skin ulcer |
| LIM068 | Norway | Sogn og F.^*^ | 21 | mar-2019 | *Salmo salar* | no | skin ulcer |
| LIM069 | Norway | Rogaland | 14 | apr-2019 | *Salmo salar* | no | skin ulcer |
| LIM070 | Norway | Rogaland | 13 | apr-2019 | *Salmo salar* | yes | mouth, gill |
| LIM071 | Norway | Troms | 22 | mar-2019 | *Salmo salar* | yes | mouth, gill |
| LIM072 | Norway | Troms | 23 | dec-2018 | *Cyclopterus*  *lumpus* | no | skin ulcer |
| LIM073 | Norway | Rogaland | 23 | dec-2018 | *Cyclopterus lumpus* | yes | skin ulcer |
| LIM074 | Norway | Rogaland | 24 | feb-2019 | *Oncorhynchus mykiss* | yes | skind ulcer |
| LIM075 | Norway | Hordaland | 24 | feb-2019 | *Oncorhynchus mykiss* | no | skind ulcer |
| LIM076 | Norway | Hordaland | 25 | may-2019 | *Salmo salar* | yes | mouth |
| LIM077 | Norway | Rogaland | 25 | may-2019 | *Salmo salar* | yes | mouth |
| LIM078 | Norway | Rogaland | 26 | jul-2019 | *Hippoglossus*  *hippoglossus* | no | gill |
| LIM079 | Norway | Hordaland | 27 | oct-2019 | *Hippoglossus hippoglossus* | no | gill |

| LIM080 | Norway Troms | 28 | feb-2020 *Clupea harengu* | *s* yes | skin ulcer |
| --- | --- | --- | --- | --- | --- |
| LIM081 | Norway Troms | 28 | feb-2020 *Clupea harengu* | *s* yes | skin ulcer |

144

145

^*^: Sogn og Fjordane

146

#### Genetic characterization

147

The *Tenacibaculum* MLST website assigned a total of 29 STs from the *Tenacibaculum* spp.

148

isolates included in this study, with 27 being new STs to the database. Two STs were

149

previously isolated from Atlantic salmon in 2010 (ST-52 and ST-53) and in 2011 (ST-52). In

150

the present study, ST-53 was isolated from a diseased Atlantic salmon farmed in Troms in

151

April 2018. ST-52 was isolated from two Atlantic salmon, in Mars 2019 in Akershus and in

152

May 2019 in Troms. (Fig 1).

153

**Fig 1. Minimum spanning tree.** Minimum spanning tree of *Tenacibaculum* strains based on 7

154

loci (*atpA*, *dnaK*, *glyA*, *gyrB*, *infB*, *rlmN* and *tgt*) using the goeBURST Full MLST algorithm.

155

The	sequence	types	(STs)	are	identified	by	a	number	(ST-,	see

156

<https://pubmlst.org/tenacibaculum/>). The circle size reflects their abundance in the data set.

157

Group founders are indicated by a yellow circle. The analysis includes 67 new isolates for the

158

database (66 STs from this study and ST-174 assigned to HFJ strain), and 18 STs already

159

160

published. The DNA relatedness is presented with emphasis on regions (A) or hosts (B).

161

Of the 27 new STs, 22 were isolated during a single outbreak of Tenacibaculosis. Five STs were

162

isolated during more than one outbreak: ST-160, ST-162, ST-107, ST-152 and ST-172.

163

ST-160 and ST-162 were associated with outbreaks of ulcerative skin disease that occurred in

164

April 2018 in Troms. Both were isolated from Atlantic salmon from the same farm during two

165

samplings separated by seven days. ST-160 was isolated from both a skin ulcer and from the

166

head kidney of the same fish. Two Atlantic salmon with skin ulcers were also infected with ST-

167

160 in another farm in Troms during the same period (April 2018).

168

ST-107 was isolated from Atlantic salmon during Tenacibaculosis outbreaks at a single site in

169

Hordaland in March, April, and November 2019. ST-107 was also isolated from a single site

170

rearing herring that presented with ulcerative disease in February 2020.

171

ST-152 was isolated from Atlantic salmon in Hordaland (April 2017, March, and April 2019),

172

in Troms (April and September 2018) and in Akershus (March 2019).

173

The most common ST found in this dataset was ST-172. It was isolated from salmon in three

174

different counties: Sogn og Fjordane (April 2018), Nordland (Mars and April 2019) and Troms

175

(April 2018, April, and May 2019). ST-172 was also isolated from rainbow trout (February

176

2019) and lumpfish in Rogaland (April 2019). This ST was associated with severe

177

tenacibaculosis.

178

#### MultiLocus Sequence Analysis (MLSA)

179

A phylogenetic tree was constructed using the 66 concatenated sequences (3894 bp) included

180

in this study. In addition, the analysis included the strain HFJ [14], 15 *Tenacibaculum* spp.

181

strains previously isolated in Norway [28], and 21 type strains in the *Tenacibaculum* genus (Fig

182

2).

183

**Fig 2. Bayesian analysis.** Bayesian phylogenetic tree of 103 *Tenacibaculum* strains based on

184

concatenated sequences of seven genes (*atpA*, *dnaK*, *glyA*, *gyrB*, *infB*, *rlmN* and *tgt*, total size:

185

3894 bp). The data set includes 66 *Tenacibaculum* strains isolated during this study (LIM

186

strains), the HFJ strain previously isolated and 36 strains from GenBank (15 from [28] (TNO

187

strains) and 21 *Tenacibaculum* Type Strains). *Kordia algicida* was used as an outgroup.

188

Posterior probabilities are indicated at each node. Fish symbols indicate the host from which

189

each strain has been isolated (LIM and TNO) and colours indicate the Norwegian administrative

190

regions, accordingly to the map. Colour dots indicate if the strains show haemolytic activity on

191

blood agar (red dot), not (blue dot) or variable (red/blue dot). Clades I-V represent the structure

192

193

of the *T. finnmarkense*/*T. dicentrarchi* group of isolates.

194

Fifty seven (57) out of the 66 strains (83 %) belong to a large and highly diversified *T. dicentrarchi*/*T.*

195

*finnmarkense* group of isolates that could be further separated into five highly supported distinct

196

clades (clades I, II, III, IV and V).

197

Clade I (the *T. finnmarkense* genomovar *ulcerans* clade) consists of 14 Norwegian

198

*Tenacibaculum* strains including strains isolated from Atlantic salmon from 1996 to 2011 (n =

199

5) and Atlantic cod in 2010 (n = 3) [20, 23, 28]. The six strains from this study were isolated

200

from Atlantic salmon (n = 5) and lumpfish (n = 1). None of these strains showed β-haemolytic

201

activity when grown on BAMA.

202

Clade II (the *T. dicentrarchi* clade) consists of eight isolates and includes the *T. dicentrarchi*

203

type strain. The six isolates from the present study were isolated from Atlantic salmon in March

204

(LIM024 and LIM025), April (LIM036) and November 2019 (LIM063) and from Atlantic

205

herring in February 2020 (LIM080 and LIM081). All six isolates presented β-haemolytic

206

activity on blood agar and were associated with clinical signs of tenacibaculosis. The clade II

207

isolates were only isolated in Hordaland and showed 100 % identity based on the seven

208

housekeeping genes (ST-107). The *Tenacibaculum* isolates TNO012 and TNO015 were

209

recovered from Atlantic cod in 2009 and 2010 and are genetically distinct from each other and

210

from all other LIM isolates in the current study.

211

Clade III (the *T. finnmarkense* genomovar *finnmarkense* clade) includes the HFJ strain, 44

212

isolates recovered during this study and seven isolates from Atlantic salmon sampled in 2010

213

and 2011 [28]. 42 isolates were collected from Atlantic salmon in Akershus, Finnmark,

214

Hordaland, Nordland and Troms. Three isolates were found in rainbow trout (LIM074) and

215

Atlantic halibut (LIM064 and LIM065) in Rogaland. All these isolates showed β-haemolytic

216

activity on blood agar except for ten isolates (TNO002, LIM006-010-017-047-049-054-055-

217

059-060-068).

218

Clade IV and clade V are both represented by a single isolate; *T. piscium* type strain (isolated

219

from an Atlantic salmon in 1998) and LIM075 (isolated from a rainbow trout in Rogaland in

220

February 2019), respectively. Like *T. piscium* [11], LIM075 did not present any β-haemolytic

221

activity on blood agar.

222

LIM057, LIM067 and LIM072 form a monophyletic group. They were isolated in Hordaland

223

(LIM057 in October 2017), Sogn og Fjordane (LIM072 in December 2018) and Troms

224

(LIM067 in March 2019), from 2 different hosts: Atlantic salmon (LIM057 and LIM067) and

225

lumpfish (LIM072). None of them showed any β-haemolytic activity on blood agar.

226

Three isolates closely related to *T. ovolyticum*. LIM058 and LIM070 were isolated from salmon

227

in Hordaland in October 2017 and April 2019, respectively. LIM073 was isolated from a

228

lumpfish in Sogn og Fjordane in December 2018. They all showed β-haemolytic activity on

229

blood agar.

230

LIM069 was isolated from an Atlantic salmon skin ulcer in Hordaland in April 2019. It did not

231

present any β-haemolytic activity on blood agar.

232

LIM078 and LIM079 are two distinct *Tenacibaculum* spp. strains isolated from farmed Atlantic

233

halibut in Rogaland. Neither strain showed β-haemolytic activity on blood agar.

234

### Discussion

235

The diagnosis of Tenacibaculosis has usually been based on the isolation of *Tenacibaculum* spp.

236

from sick fish followed by morphological, biochemical, and serological characterization of the

237

bacteria and analysis of their antimicrobial susceptibility profiles [8]. MultiLocus Sequence

238

Analysis (MLSA) has, however, been increasingly used for bacterial genotyping [17, 23, 28,

239

36, 37] and has become an important tool for diagnosis and studies of epizootics. The results

240

from the current study confirm the large diversity of *Tenacibaculum* spp. strains present in fish

241

farms along the Norwegian coast and their association with ulcerative disease outbreaks.

242

**High diversity of Norwegian *Tenacibaculum* spp. strains**

243

This study presents the genotyping of 66 *Tenacibaculum* strains isolated from April 2017 to

244

February 2020 in Norway. Phylogenetic analyses show that 57 strains belong to a major lineage

245

structured into five different clades consisting of *T. finnmarkense* genomovar *ulcerans* Clade I,

246

*T. dicentrarchi* Clade II, *T. finnmarkense* genomovar *finnmarkense* Clade III, *Tenacibaculum*

247

*piscium* Clade IV, and a putative new species Clade V (Fig 2). This is in accordance with

248

previous phylogenetic analyses of *Tenacibaculum* spp. isolated from farmed fishes in Norway

249

[11, 23, 27]. Several strains are not closely related to any *Tenacibaculum* spp. type strains

250

(LIM078 and LIM079 isolated from Atlantic halibut in Rogaland, LIM069 isolated from

251

Atlantic salmon in Hordaland; LIM057, LIM067 and LIM072 isolated from Atlantic salmon

252

and lumpfish in Troms, Hordaland, and Sogn og Fjordane). Nevertheless, further identification

253

steps must be undertaken to conclude whether these new strains represent novel *Tenacibaculum*

254

species.

255

Altogether, these results confirm the large *Tenacibaculum* diversity in Norway, and suggest

256

that other strains have yet to be identified.

257

The phylogenetic analysis clearly showed that most of the isolates recovered from Atlantic

258

salmon	suffering	tenacibaculosis	belong	to	the	species	*T. finnmarkense*	genomovar

259

*finnmarkense* and have β-haemolytic activity (except for five STs clustering in a strongly

260

supported clade). The *T. dicentrarchi* strain isolated in Hordaland (ST-107, β-haemolytic) was

261

associated with tenacibaculosis and mortality in both Atlantic salmon and Atlantic herring. The

262

pathogenicity of *T. dicentrarchi* strains has been already described in several fish species

263

including European sea bass, wrasse, Atlantic cod, and Atlantic salmon from Chile [13, 28, 37].

264

Olsen et al. suggested that three of the four *T. dicentrarchi* strains isolated from asymptomatic

265

salmon could have a higher pathogenicity towards non-salmonids species [23]. The severe

266

symptoms associated with ST-107 (LIM 024-025-036-063-080-081) suggests pathogenicity

267

towards Atlantic salmon and indicate that strains pathogenic to salmon may also occur in

268

Norway. The three strains closely related to *T. ovolyticum* were associated with ulcers and

269

showed β-haemolytic activities when grown on blood agar plates. Even though *T. ovolyticum*

270

is already known for its ability to dissolve halibut eggshell, which could lead to the death of the

271

embryo [9, 38], it is to our knowledge the first report of pathogenicity of *T. ovolyticum* toward

272

Atlantic salmon and lumpfish. Nevertheless, it is uncommon and therefore unlikely to be a

273

major threat to aquaculture.

274

β-haemolytic activity is commonly associated with pathogenic bacteria [39], but a non-

275

haemolytic strain in this data set is also associated with tenacibaculosis (ST-160, Clade III).

276

Overall, non-haemolytic *Tenacibaculum* strains seem to be less pathogenic and may not lead to

277

tenacibaculosis outbreaks. Indeed, when associated with outbreaks, Clade I strains were

278

isolated together with other haemolytic strains (LIM003, LIM004, LIM012, LIM016). Some

279

Clade I strains were sporadically associated with ulcer symptoms (LIM066 for instance). This

280

reinforces the need for the use of blood-supplemented culture media in the field. Estimating as

281

early as possible the virulence of a strain is necessary in order to implement rapid sanitary

282

measures.

283

#### Presence of clinically relevant ubiquitous strains

284

*Tenacibaculum finnmarkense* isolates from clades I and III are present all along the Norwegian

285

coast, from Akershus in the southeast to Finnmark in the north. It is interesting to note that all

286

the STs from Clade III except one (LIM064, isolated in Rogaland) have been isolated at least

287

once in Troms or Finnmark. To note, *Tenacibaculum finnmarkense* has also been isolated

288

outside of Norway, from Chilean Atlantic salmon, coho salmon, and rainbow trout

289

(*Oncorhynchus kisutch*) [27, 36, 40].	*Tenacibaculum dicentrarchi strains* and the strains

290

closely related to *T. ovolyticum* were only isolated in the western part of Norway. The reason

291

why *T. dicentrarchi* and *T. ovolyticum* seem to be mostly present in western Norway is

292

unknown but may be linked to several factors such as higher seawater temperature in western

293

Norway compared to northern Norway.

294

93 % of the STs described herein were new to the MLST database. Only two STs (ST-52 and

295

ST-53) were previously isolated from Atlantic salmon in 2010 and 2011 [23]. The majority of

296

these new STs (81 %) were recovered from a single outbreak event and therefore were

297

associated with a single locality and host. This is in accordance with previous results that

298

showed that the overall lack of clonality and host specificity among the Norwegian

299

*Tenacibaculum* isolates indicated that Tenacibaculosis infections arise as local epidemics

300

involving multiple strains [23]. However, several STs were isolated from different localities

301

and /or from different hosts and are therefore of extreme importance for the aquaculture. ST-

302

152 is associated with Tenacibaculosis outbreaks in farmed Atlantic salmon in three different

303

counties: Troms, Hordaland and Akershus. ST-172 is the strain with the widest distribution,

304

from Troms in the north, to Rogaland in the south. This ST, always associated with severe

305

tenacibaculosis, might have a wider tolerance to different seawater conditions (salinity and

306

temperature) than *T. dicentrarchi*. More importantly, both STs were isolated throughout the

307

sampling period, from April 2017 to May 2019. We isolated ST-172 again in March 2020

308

during an outbreak in an Atlantic salmon farm in Troms. ST-172 has been isolated from three

309

different fish species: Atlantic salmon, rainbow trout and lumpfishes. Even though it is

310

uncommon, the ability of some *Tenacibaculum* strains to infect several fish species has been

311

shown already [23, 28], but they were limited to a restricted geographic area. Likewise, the *T.*

312

*dicentrarchi* related ST-107 isolated from both Atlantic salmon and Atlantic herring, was found

313

only in Hordaland. But ST-172 and ST-152 present the ability to spread within a broad

314

geographic area. How they spread out along the Norwegian coast and how they maintain

315

themselves in the environment between outbreaks remain unknown, but several hypotheses can

316

be made. Avendaño-Herrera et al. suggested that seawater is not an important route of

317

transmission for *T. maritimum* and hypothesised that bacteria need to be attached to particle or

318

substrate such as sediments or fish mucus to be maintained until favourable conditions occur

319

[41]. Levipan et al. showed that all the *T. dicentrarchi* strains in their study were able to adhere

320

to and form biofilms on polystyrene surfaces [42]. Survival of *T. maritimum* in fish mucus [43]

321

suggests that bacteria can colonize and spread together with its hosts. Fish movements at

322

different life stages can therefore be a spreading route for their associated bacteria. Furthermore,

323

the transport of pathogens by fish ectoparasites have already been reported. For instance, the

324

sea lice *Lepeophtheirus salmonis*, a common salmonid associated ectoparasite, can carry

325

several micro-organisms such as *Aeromonas salmonicida* [44] and *T. maritimum* [45]. Thus,

326

studies assessing the transport of *Tenacibaculum* sp. need to be implemented.

327

### Conclusion

328

MultiLocus Sequence Typing is a powerful tool for studies of epizootics caused by bacteria and other pathogens. It

329

is highly efficient to get an overview of a bacterial community and to separate strains with

330

putative different pathogenicity. Overall, the results from this study consolidate the conclusions

331

of previous studies and confirm the high diversity of the *Tenacibaculum* spp. strains infecting

332

farmed fishes in Norway. This high diversity of *Tenacibaculum* spp. strains and their broad

333

geographical distribution is a major challenge for aquaculture, not only in Norway but also

334

globally~~. Attempts to tackle some of these major outbreaks by focusing on vaccines have so~~

~~335~~

~~far been unsuccessful, which increases the importance of knowledge about reservoirs,~~

~~336~~

~~transmission routes and virulence differences.~~

337

### Acknowledgments

338

We are deeply thankful to all the farmers and veterinarians from the Norwegian fish farms who

339

greatly helped in the sampling. We also want to thank Lars Are Hamre (Sea Lice Research

340

Centre, University of Bergen) and Kjetil Solheim (Fish health manager of Sterling White

341

Halibut AS) who helped to obtain Atlantic salmon from Hordaland and Atlantic halibuts from

342

Rogaland, respectively.

343

### References

344

345

346

347

348

349

350

351

352

353

354

355

356

357

1. Toranzo AE, Magariños B, Romalde JL. A review of the main bacterial fish diseases in mariculture systems. Aquaculture. 2005;246(1):37-61. doi: [https://doi.org/10.1016/j.aquaculture.2005.01.002.](https://doi.org/10.1016/j.aquaculture.2005.01.002)
2. Avendaño-Herrera R, Toranzo AE, Magariños B. Tenacibaculosis infection in marine fish caused by Tenacibaculum maritimum: a review. Diseases of aquatic organisms. 2006;71(3):255-66. doi: 10.3354/dao071255. PubMed PMID: 17058606.
3. Campbell AC, Buswell J. An investigation into the bacterial aetiology of@ black patch necrosi“ in Dover sole, Solea solea L. Journal of Fish Diseases. 1982;5:495-508.
4. Devesa S, Barja JL, Toranzo AE. Ulcerative skin and fin lesions in reared turbot, Scophthalmus maximus (L.). [Oxford, England] :1989. p. 323-33.
5. Handlinger J, Soltani M, Percival S. The pathology of Flexibacter maritimus in aquaculture species in Tasmania, Australia. [Oxford, England] :1997. p. 159-68.
6. Mcvicar A, White PG. Fin and skin necrosis of cultivated Dover sole Solea solea (L.). Journal of Fish Diseases. 1979;2:557-62.

358

359

360

361

362

363

364

365

366

367

368

369

370

371

372

373

374

375

376

377

378

379

380

381

382

383

384

385

386

387

388

389

390

391

392

393

394

395

396

397

398

399

400

401

402

403

404

405

406

407

408

409

7. Wakabayashi H, Hikida M, Masumura K, editors. Flexibacter maritimus sp. nov., a pathogen of marine fishes1986.
8. Fernández-Álvarez C, Santos Y. Identification and typing of fish pathogenic species of the genus Tenacibaculum. Appl Microbiol Biotechnol. 2018;102(23):9973-89. Epub 2018/10/05. doi: 10.1007/s00253-018-9370-1. PubMed PMID: 30291367.
9. Hansen GH, Bergh O, Michaelsen J, Knappskog D. Flexibacter ovolyticus sp. nov., a pathogen of eggs and larvae of Atlantic halibut, Hippoglossus hippoglossus L. Int J Syst Bacteriol. 1992;42(3):451-8. Epub 1992/07/01. doi: 10.1099/00207713-42-3-451. PubMed PMID: 1503974.
10. Kim Y-O, Park S, Nam B-H, Jung Y-T, Kim D-G, Jee Y-J, et al. Tenacibaculum halocynthiae sp. nov., a member of the family Flavobacteriaceae isolated from sea squirt Halocynthia roretzi. Antonie van Leeuwenhoek. 2013;103(6):1321-7. doi: 10.1007/s10482-013-9913-5.
11. Olsen AB, Spilsberg B, Nilsen HK, Lagesen K, Gulla S, Avendaño-Herrera R, et al. Tenacibaculum piscium sp. nov., isolated from skin ulcers of sea-farmed fish, and description of Tenacibaculum finnmarkense sp. nov. with subdivision into genomovars finnmarkense and ulcerans. Int J Syst Evol Microbiol. 2020. doi: [https://doi.org/10.1099/ijsem.0.004501.](https://doi.org/10.1099/ijsem.0.004501)
12. Park S, Choi J, Choi SJ, Yoon J-H. Tenacibaculum insulae sp. nov., isolated from a tidal flat. Int J Syst Evol Microbiol. 2018;68(1):228-33. Epub 2017/11/17. doi: 10.1099/ijsem.0.002487. PubMed PMID: 29148365.
13. Piñeiro-Vidal M, Gijón D, Zarza C, Santos Y. Tenacibaculum dicentrarchi sp. nov., a marine bacterium of the family Flavobacteriaceae isolated from European sea bass. Int J Syst Evol Microbiol. 2012;62(Pt 2):425-9. Epub 2011/04/05. doi: 10.1099/ijs.0.025122-0. PubMed PMID: 21460137.
14. Småge SB, Brevik OJ, Duesund H, Ottem KF, Watanabe K, Nylund A. Tenacibaculum finnmarkense sp. nov., a fish pathogenic bacterium of the family Flavobacteriaceae isolated from Atlantic salmon. Antonie Van Leeuwenhoek. 2016;109(2):273-85. Epub 2015/12/15. doi: 10.1007/s10482-015-0630-0. PubMed PMID: 26662517; PubMed Central PMCID: PMCPMC4751178.
15. Avendaño-Herrera R, Magariños B, López-Romalde S, Romalde JL, Toranzo AE. Phenotypic characterization and description of two major O-serotypes in Tenacibaculum maritimum strains from marine fishes. Dis Aquat Organ. 2004;58(1):1-8. Epub 2004/03/25. doi: 10.3354/dao058001. PubMed PMID: 15038445.
16. Chen ME, Henry-Ford D, Groff JM. Isolation and Characterization of Flexibacter maritimus from Marine Fishes of California. Journal of Aquatic Animal Health. 1995;7(4):318-26. doi: 10.1577/1548-8667(1995)007<0318:IACOMF>2.3.CO;2.
17. Frisch K, Småge SB, Brevik OJ, Duesund H, Nylund A. Genotyping of Tenacibaculum maritimum isolates from farmed Atlantic salmon in Western Canada. J Fish Dis. 2018;41(1):131-7. Epub 2017/07/27. doi: 10.1111/jfd.12687. PubMed PMID: 28744871.
18. Ostland VE, Morrison D, Ferguson HW. Flexibacter Maritimus Associated with a Bacterial Stomatitis in Atlantic Salmon Smolts Reared in Net-Pens in British Columbia. Journal of Aquatic Animal Health. 1999;11(1):35-44. doi: 10.1577/1548-8667(1999)011<0035:FMAWAB>2.0.CO;2.
19. Suzuki M, Nakagawa Y, Harayama S, Yamamoto S. Phylogenetic analysis and taxonomic study of marine Cytophaga-like bacteria: proposal for Tenacibaculum gen. nov. with Tenacibaculum maritimum comb. nov. and Tenacibaculum ovolyticum comb. nov., and description of Tenacibaculum mesophilum sp. nov. and Tenacibaculum amylolyticum sp. nov. Int J Syst Evol Microbiol. 2001;51(Pt 5):1639-52. Epub 2001/10/12. doi: 10.1099/00207713-51-5-1639. PubMed PMID: 11594591.
20. Olsen AB, Nilsen H, Sandlund N, Mikkelsen H, Sorum H, Colquhoun DJ. Tenacibaculum sp. associated with winter ulcers in sea-reared Atlantic salmon Salmo salar. Dis Aquat Organ. 2011;94(3):189-99. Epub 2011/07/28. doi: 10.3354/dao02324. PubMed PMID: 21790066.
21. Småge SB, Brevik OJ, Frisch K, Watanabe K, Duesund H, Nylund A. Concurrent jellyfish blooms and tenacibaculosis outbreaks in Northern Norwegian Atlantic salmon (Salmo salar) farms. PLoS One. 2017;12(11):e0187476. Epub 2017/11/03. doi: 10.1371/journal.pone.0187476. PubMed PMID: 29095885; PubMed Central PMCID: PMCPMC5667831.
22. Småge SB, Frisch K, Brevik ØJ, Watanabe K, Nylund A. First isolation, identification and characterisation of Tenacibaculum maritimum in Norway, isolated from diseased farmed sea lice

410

411

412

413

414

415

416

417

418

419

420

421

422

423

424

425

426

427

428

429

430

431

432

433

434

435

436

437

438

439

440

441

442

443

444

445

446

447

448

449

450

451

452

453

454

455

456

457

458

459

460

cleaner fish Cyclopterus lumpus L. Aquaculture. 2016;464:178-84. doi: 10.1016/j.aquaculture.2016.06.030.

23. Olsen AB, Gulla S, Steinum T, Colquhoun DJ, Nilsen HK, Duchaud E. Multilocus sequence analysis reveals extensive genetic variety within Tenacibaculum spp. associated with ulcers in sea- farmed fish in Norway. Vet Microbiol. 2017;205:39-45. Epub 2017/06/18. doi: 10.1016/j.vetmic.2017.04.028. PubMed PMID: 28622859.
24. Klakegg Ø, Abayneh T, Fauske AK, Fülberth M, Sørum H. An outbreak of acute disease and mortality in Atlantic salmon (Salmo salar) post-smolts in Norway caused by Tenacibaculum dicentrarchi. J Fish Dis. 2019;42(6):789-807. Epub 2019/03/21. doi: 10.1111/jfd.12982. PubMed PMID: 30893484.
25. Lunder T, Evensen O, Holstad G, Hastein T, Evensen O. 'Winter ulcer' in the Atlantic salmon Salmo salar. Pathological and bacteriological investigations and transmission experiments. Diseases of Aquatic Organisms. 1995;23(1):39-49. doi: 10.3354/dao023039.
26. Salte R, Rørvik KA, Reed E, Norberg K. Winter ulcers of the skin in Atlantic salmon, Salmo salar L.: pathogenesis and possible aetiology. Journal of Fish Diseases. 1994;17(6):661-5. doi: 10.1111/j.1365-2761.1994.tb00265.x.
27. Bridel S, Olsen AB, Nilsen H, Bernardet JF, Achaz G, Avendaño-Herrera R, et al. Comparative genomics of *Tenacibaculum dicentrarch*i and “*Tenacibaculum finnmarkense*” highlights intricate evolution of fish-pathogenic species. Genome Biology and Evolution. 2018:evy020-evy. doi: 10.1093/gbe/evy020.
28. Habib C, Houel A, Lunazzi A, Bernardet JF, Olsen AB, Nilsen H, et al. Multilocus sequence analysis of the marine bacterial genus Tenacibaculum suggests parallel evolution of fish pathogenicity and endemic colonization of aquaculture systems. Appl Environ Microbiol. 2014;80(17):5503-14. Epub 2014/06/29. doi: 10.1128/AEM.01177-14. PubMed PMID: 24973065; PubMed Central PMCID: PMCPMC4136090.
29. Giovannoni SJ, Rappé MS, Vergin KL, Adair NL. 16S rRNA genes reveal stratified open ocean bacterioplankton populations related to the Green Non-Sulfur bacteria. Proc Natl Acad Sci U S A. 1996;93(15):7979-84. Epub 1996/07/23. doi: 10.1073/pnas.93.15.7979. PubMed PMID: 8755588; PubMed Central PMCID: PMCPMC38860.
30. Altschul SF, Gish W, Miller W, Myers EW, Lipman DJ. Basic local alignment search tool. J Mol Biol. 1990;215(3):403-10. Epub 1990/10/05. doi: 10.1016/s0022-2836(05)80360-2. PubMed PMID: 2231712.
31. Francisco AP, Bugalho M, Ramirez M, Carriço JA. Global optimal eBURST analysis of multilocus typing data using a graphic matroid approach. BMC Bioinformatics. 2009;10:152-. doi: 10.1186/1471-2105-10-152. PubMed PMID: 19450271.
32. Feil EJ, Li BC, Aanensen DM, Hanage WP, Spratt BG. eBURST: inferring patterns of evolutionary descent among clusters of related bacterial genotypes from multilocus sequence typing data. J Bacteriol. 2004;186(5):1518-30. Epub 2004/02/20. doi: 10.1128/jb.186.5.1518-1530.2004. PubMed PMID: 14973027; PubMed Central PMCID: PMCPMC344416.
33. Nicholas KBaHBN, editor Nicholas, Karen B. and Hugh B. Nicholas. “GeneDoc: a tool for editing and annotating multiple sequence alignments.” (1997).1997 1997.
34. Tanabe AS. Kakusan4 and Aminosan: two programs for comparing nonpartitioned, proportional and separate models for combined molecular phylogenetic analyses of multilocus sequence data. Mol Ecol Resour. 2011;11(5):914-21. Epub 2011/05/20. doi: 10.1111/j.1755- 0998.2011.03021.x. PubMed PMID: 21592310.
35. Ronquist F, Huelsenbeck JP. MrBayes 3: Bayesian phylogenetic inference under mixed models. Bioinformatics. 2003;19(12):1572-4. Epub 2003/08/13. doi: 10.1093/bioinformatics/btg180. PubMed PMID: 12912839.
36. Avendaño-Herrera R, Collarte C, Saldarriaga-Córdoba M, Irgang R. New salmonid hosts for Tenacibaculum species: Expansion of tenacibaculosis in Chilean aquaculture. J Fish Dis. 2020. Epub 2020/07/15. doi: 10.1111/jfd.13213. PubMed PMID: 32662133.

461

462

463

464

465

466

467

468

469

470

471

472

473

474

475

476

477

478

479

480

481

482

483

484

485

486

487

488

489

490

491

492

37. Avendaño-Herrera R, Irgang R, Sandoval C, Moreno-Lira P, Houel A, Duchaud E, et al. Isolation, Characterization and Virulence Potential of Tenacibaculum dicentrarchi in Salmonid Cultures in Chile. Transbound Emerg Dis. 2016;63(2):121-6. Epub 2016/01/11. doi: 10.1111/tbed.12464. PubMed PMID: 26749435.
38. Bergh Ø, Hansen GH, Taxt RE. Experimental infection of eggs and yolk sac larvae of halibut, Hippoglossus hippoglossus L. Journal of Fish Diseases. 1992;15(5):379-91. doi: 10.1111/j.1365- 2761.1992.tb01237.x.
39. Sum R, Swaminathan M, Rastogi SK, Piloto O, Cheong I. Beta-Hemolytic Bacteria Selectively Trigger Liposome Lysis, Enabling Rapid and Accurate Pathogen Detection. ACS Sens. 2017;2(10):1441- 51. Epub 2017/09/21. doi: 10.1021/acssensors.7b00333. PubMed PMID: 28929742.
40. Grothusen H, Castillo A, Henríquez P, Navas E, Bohle H, Araya C, et al. First Complete Genome Sequence of Tenacibaculum dicentrarchi, an Emerging Bacterial Pathogen of Salmonids. Genome Announc. 2016;4(1). Epub 2016/02/20. doi: 10.1128/genomeA.01756-15. PubMed PMID: 26893432; PubMed Central PMCID: PMCPMC4759079.
41. Avendaño-Herrera R, Irgang R, Magariños B, Romalde JL, Toranzo AE. Use of microcosms to determine the survival of the fish pathogen Tenacibaculum maritimum in seawater. Environ Microbiol. 2006;8(5):921-8. Epub 2006/04/21. doi: 10.1111/j.1462-2920.2005.00981.x. PubMed PMID: 16623748.
42. Levipan HA, Irgang R, Tapia-Cammas D, Avendano-Herrera R. A high-throughput analysis of biofilm formation by the fish pathogen Tenacibaculum dicentrarchi. J Fish Dis. 2019;42(4):617-21. Epub 2019/01/22. doi: 10.1111/jfd.12949. PubMed PMID: 30664803.
43. Magarinos B, Pazos F, Santos Y, Romalde JL, Toranzo AE. Response of Pasteurella piscicida and Flexibacter maritimus to skin mucus of marine fish. Diseases of Aquatic Organisms. 1995;21(2):103-8.
44. Nese L, Enger Ø. Isolation of Aeromonas salmonicida from salmon lice Lepeophtheirus salmonis and marine plankton. Diseases of Aquatic Organisms - DISEASE AQUAT ORG. 1993;16:79-81. doi: 10.3354/dao016079.
45. Barker DE, Braden LM, Coombs MP, Boyce B. Preliminary studies on the isolation of bacteria from sea lice, Lepeophtheirus salmonis, infecting farmed salmon in British Columbia, Canada. Parasitol Res. 2009;105(4):1173-7. Epub 2009/07/01. doi: 10.1007/s00436-009-1523-9. PubMed PMID: 19565269.

493

### Supporting information

494

**S1 Table. Allelic profiles.** Sequence type (ST) for 67 *Tenacibaculum* strains and unique allelic

495

identifiers for the seven loci considered in the MLST scheme assigned by the established

496

*Tenacibaculum* MLST website (<https://pubmlst.org/tenacibaculum/>)

497

S2 Table. GenBank accession numbers of the sequences produced in this study.

Fig1

[Click here to aceess/dCflYnload;Figure;Fig1.tif](https://www.editorialmanager.com/pone/download.aspx?id=29338566&amp;guid=a2fe9b37-1357-41e1-b068-56b977f13690&amp;scheme=1) [*i*](https://www.editorialmanager.com/pone/download.aspx?id=29338566&amp;guid=a2fe9b37-1357-41e1-b068-56b977f13690&amp;scheme=1)

A - Regions

•

•

_-.. ,....,

....

•	""IC!f.!

.

1	...l•or.Q

.........._,,, ,

......,_..,..,.I

•_ ...r.Qnt

###### B - Hosts

••

·.---rt.....

.

- e

# .... . •

• •..·•• •

••

............,. ""'""'

##### ....

_ n111

•• •

• 0..---...:JO't,1

•	*..,- ...atN*	•

•

Fig2

Click he1e to f CCtss/downlod;Figu1t:Flg2.tif .&

*"""·*•..,.'4

•s •

•

-....-_

'_.."."_

.. .. 1·4 -o ... ........

·-

.,..,._.....

.: ·	-

•-

- ·-·

-

•

## -··

,_ ,,.._

l.J	.._

,,,_

##### u

. -

H -

~~...~~

---·

S1 table

[Click here to access/download](https://www.editorialmanager.com/pone/download.aspx?id=29338586&amp;guid=b11b8b58-567b-4402-8ef6-2ac544af3715&amp;scheme=1)

[**Supporting Information** S1 Table.docx](https://www.editorialmanager.com/pone/download.aspx?id=29338586&amp;guid=b11b8b58-567b-4402-8ef6-2ac544af3715&amp;scheme=1)

S2 table

[Click here to access/download](https://www.editorialmanager.com/pone/download.aspx?id=29338587&amp;guid=a49f09d1-2e64-4935-9458-50e30364b32f&amp;scheme=1)

[**Supporting Information** S2 Table.docx](https://www.editorialmanager.com/pone/download.aspx?id=29338587&amp;guid=a49f09d1-2e64-4935-9458-50e30364b32f&amp;scheme=1)
